# Sirtuin 6 inhibits epithelial to mesenchymal transition during idiopathic pulmonary fibrosis via inactivating TGF-β1/Smad3 signaling

**DOI:** 10.18632/oncotarget.17723

**Published:** 2017-05-09

**Authors:** Kunming Tian, Panpan Chen, Zhiping Liu, Shutian Si, Qian Zhang, Yong Mou, Lianyong Han, Qin Wang, Xue Zhou

**Affiliations:** ^1^ Department of Occupational and Environmental Health, Key Laboratory of Environment and Health, Ministry of Education & Ministry of Environmental Protection, State Key Laboratory of Environmental Health (Incubating), School of Public Health, Tongji Medical College, Huazhong University of Science and Technology, Wuhan, China; ^2^ Vascular Biology Center, Medical College of Georgia, Augusta University, Augusta, USA; ^3^ The Center for Biomedical Research, Tongji Hospital, Tongji Medical College, Huazhong University of Science and Technology, Wuhan, China; ^4^ Department of Respiratory and Critical Care Medicine, Key Laboratory of Pulmonary Diseases, Chinese Ministry of Health, Tongji Hospital, Tongji Medical College, Huazhong University of Science and Technology, Wuhan, China

**Keywords:** Sirt6, epithelial to mesenchymal transition, TGF-β1/Smad3, idiopathic pulmonary fibrosis, adeno-associated virus

## Abstract

Sirt6 which is implicated in the control of aging, cancer, and metabolism, has been shown to have anti-fibrosis function in heart and liver. However, whether Sirt6 inhibits idiopathic pulmonary fibrosis remains elusive. Epithelial to mesenchymal transition has been found to be involved in the pathogenesis of idiopathic pulmonary fibrosis. In the present study, forced expression of Sirt6 significantly abrogated TGF-β1-induced epithelial to mesenchymal transition-like phenotype and cell behaviors in A549 cells. Additionally, activation of TGF-β1/Smad3 signaling pathway and binding of Smad3-Snail1 were ameliorated by overexpression of wild-type Sirt6 but not mutant Sirt6 (H133Y) without histone deacetylase activity. Meanwhile, upregulation of epithelial to mesenchymal transition-related transcription factors by TGF-β1 were also restored by overexpression of wild-type Sirt6 but not mutant Sirt6. Furthermore, *in vivo* study showed that lung targeted delivery of Sirt6 using adeno-associated virus injection blunted bleomycin-induced pulmonary epithelial to mesenchymal transition and fibrosis. Overall, our findings unravel that Sirt6 acts as a key modulator in epithelial to mesenchymal transition process, suggesting Sirt6 may be an attractive potential therapeutic target for idiopathic pulmonary fibrosis.

## INTRODUCTION

Idiopathic pulmonary fibrosis (IPF) is an irreversible, progressive, and short mean survival interstitial lung disease, characterized by excessive extracellular matrix (ECM) deposition and lung architecture damage [1]. Patients with IPF have median survival of 2 to 3 years after diagnosis and the incidence of IPF continues to rise [2]. However, there is no effective therapy so far. Thus, it is urgent to mechanistically understand the pathogenesis of IPF to develop new treatments for this fatal disease.

It has been well established that myofibroblasts, characterized by increased expression of α-smooth muscle actin (α-SMA) and synthesis of a large amount of ECM components, are the main effector cells in the pathogenesis of lung fibrosis [3, 4]. Damping the formation and activation of myofibroblasts has been recognized as a promising therapeutic approach for prevention and treatment of lung fibrosis [5]. Three mechanisms likely contribute to accumulation of myofibroblasts: (1) activation of resident fibroblasts in response to lung injury [6]; (2) circulating bone marrow origin of mesenchymal fibrocytes [7]; (3) differentiation of lung alveolar epithelial cells (AECs) into myofibroblasts via epithelial to mesenchymal transition (EMT) [8, 9].

EMT is a process in which epithelial cells lose epithelial morphology and biomarkers as well as gain mesenchymal phenotype [10, 11]. Accumulating evidence has demonstrated that EMT is involved in embryonic development [12], initiation of invasion [13], and multiple tissue fibrosis, including lung [8, 14]. Lineage tracing in animal lung fibrosis model uncovered that approximately one-third of myofibroblasts were originated from lung epithelium [8, 14]. In addition, EMT phenotype were widely observed in lung biopsy from IPF patients [8, 15]. Therefore, determining the key molecules which regulate EMT or dedifferentiate mesenchymal cells to epithelial cells represents an attractive therapeutic strategy to suppress or reverse IPF.

Sirt6 is a member of the sirtuin family of NAD-dependent histone deacetylases. It has a wide range of role in aging, cancer, and metabolism [16], and it also exerts a protective role in cardiac and liver fibrosis. Both of the mice with cardiac-specific depletion of Sirt6 and crossbred Sirt6 knockout mice exhibited increased cardiac fibrosis, while Sirt6 transgenic mice showed weaken cardiac fibrosis [17]. In addition, we previously found that knockdown of Sirt6 in primary cardiac fibroblasts resulted in augmented differentiation of cardiac fibroblasts to myofibroblasts and cardiac fibrosis [18]. Moreover, Sirt6 mutant mice also developed liver fibrosis [19].

Although Sirt6 has been recognized as a key modulator in cardiac and liver fibrosis, to the best of our knowledge, whether Sirt6 is involved in pulmonary EMT process has yet to be identified. In the present study, we aimed to investigate the role of Sirt6 in EMT process and EMT-related pulmonary fibrosis in both TGF-β1-induced A549 cells and bleomycin-induced experimental model. We found that TGF-β1-induced EMT phenotype and EMT-like cell behaviors were repressed by Sirt6 overexpression, at least partially through inactivation of TGF-β1/Smad3 signaling pathway.

## RESULTS

### Sirt6 is upregulated in TGF-β1-treated A549 cells and bleomycin-injured mice

**Figure 1:**
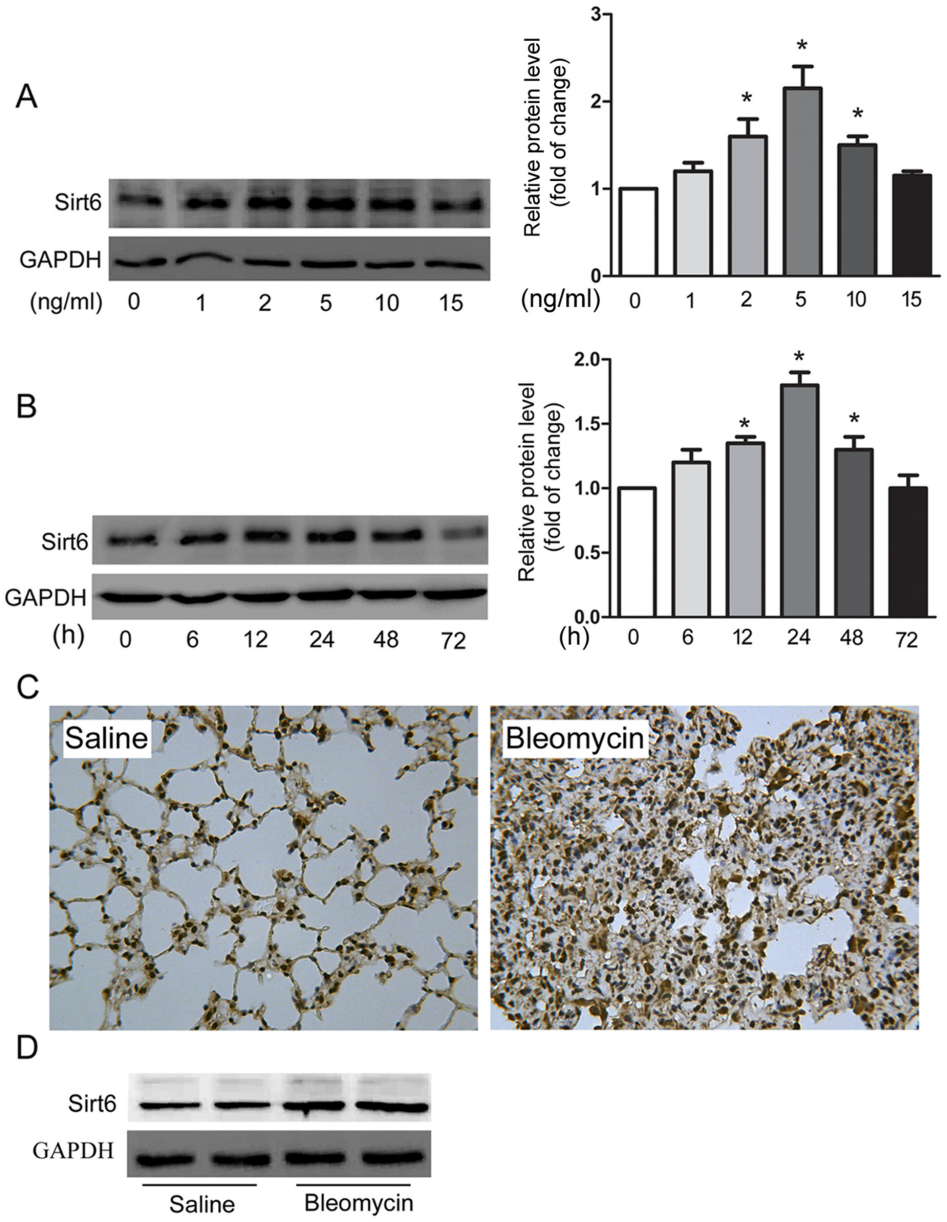
Sirt6 is upregulated in *in vitro* and *in vivo* models of EMT A549 cells were treated with indicated concentrations of TGF-β1 for 24 h **(A)** or with 5 ng/ml of TGF-β1 for indicated time **(B)**, and Western blot analysis of Sirt6 was performed. **(C-D)** IHC (C) and Western blot analysis (D) of Sirt6 in lungs from C57BL/6 mice sacrificed 21d after 2 mg/kg bleomycin treatment (magnification, 200×). n = 6 mice per group. ^*^P < 0.05.

We first investigated expression of Sirt6 in both cultured and experimental EMT models. As shown in Figure 1A and 1B, TGF-β1 upregulated the protein level of Sirt6 in a dose-and time-dependent manner in A549 cells. In the well-established experimental fibrosis model, Sirt6 was markedly increased in mouse lung in response to bleomycin treatment, as evidenced by immunohistochemistry (IHC) and Western blot analysis (Figure 1C and 1D). Collectively, these results imply that Sirt6 could be associated with the pathogenesis of EMT.

### Sirt6 is sufficient to restore EMT phenotype in A549 cells

**Figure 2:**
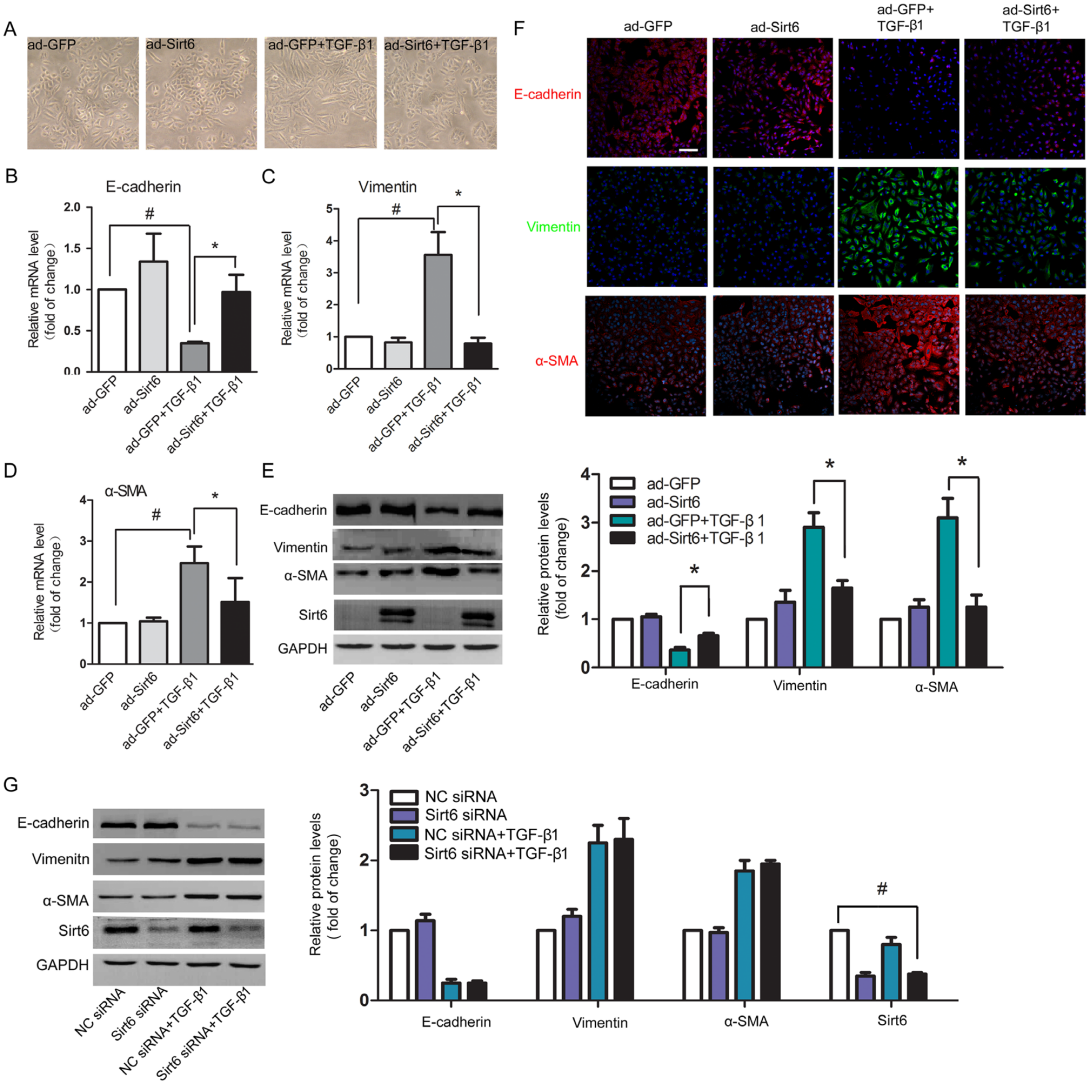
Sirt6 reverses TGF-β1-induced EMT in A549 cells A549 cells were transfected with ad-GFP or ad-Sirt6 in the absence or presence of TGF-β1 (5 ng/ml) for 24h. **(A)** Morphological changes of A549 cells (magnification, 200×). **(B-D)** Real-time PCR analysis of the mRNA levels of E-cadherin (B), vimentin (C), and α-SMA (D). **(E)** Western blot analysis of the protein levels of E-cadherin, vimentin, α-SMA and Sirt6. **(F)** Representative images of the immunofluorescent staining of E-cadherin, vimentin, and α-SMA. Scale bar, 100 μm. **(G)** A549 cells were transfected with NC siRNA or Sirt6 siRNA in the absence or presence of TGF-β1 (5 ng/ml) for 24h, and the protein levels E-cadherin, vimentin, and α-SMA of were determined by Western blot. All data represent the means ± SEM of three independent experiments. Compared with ad-GFP+TGF-β1 group, ^*^P < 0.05; ^#^*P* < 0.01.

The potential role of Sirt6 in pulmonary EMT was next examined. Sirt6 expression was strongly upregulated after ad-Sirt6 transfection (Figure 2E). As shown in Figure 2A, A549 cells exhibited epithelial cobblestone phenotype under physiologic condition. After TGF-β1 stimulation, the cells were converted to mesenchymal phenotype characterized by spindle-shape. Sirt6 alone had no effect on the morphology of the cells. However, forced expression of Sirt6 rescued TGF-β1-induced EMT characteristic (Figure 2A). In agreement with the change in cellular appearance, TGF-β1-elicited decrease of the epithelial marker E-cadherin and increase of the mesenchymal markers vimentin and α-SMA at both of the mRNA (Figures 2B-2D) and protein levels (Figure 2E) were restored by Sirt6 overexpression. Moreover, Sirt6 overexpression also inhibited EMT process in primary mouse alveolar epithelial cells (Supplementary Figure 1). This notion was further confirmed by immunofluorescent analysis (Figure 2F). To further verify the negative regulatory role of Sirt6 in EMT, we efficiently knocked down Sirt6 using siRNA targeted Sirt6. Western blot were performed to evaluate the knockdown efficiency of three independent siRNAs, marked S1, S2, and S3, respectively. The siRNA S1 achieved the best silencing efficiency (Supplementary Figure 2) and was used in the following studies. Unexpectedly, loss of Sirt6 failed to impact EMT markers (Figure 2G). Taken together, these results provided evidences that gain but not loss of Sirt6 regulates EMT process.

### Sirt6 regulates EMT-like cell behaviors

**Figure 3:**
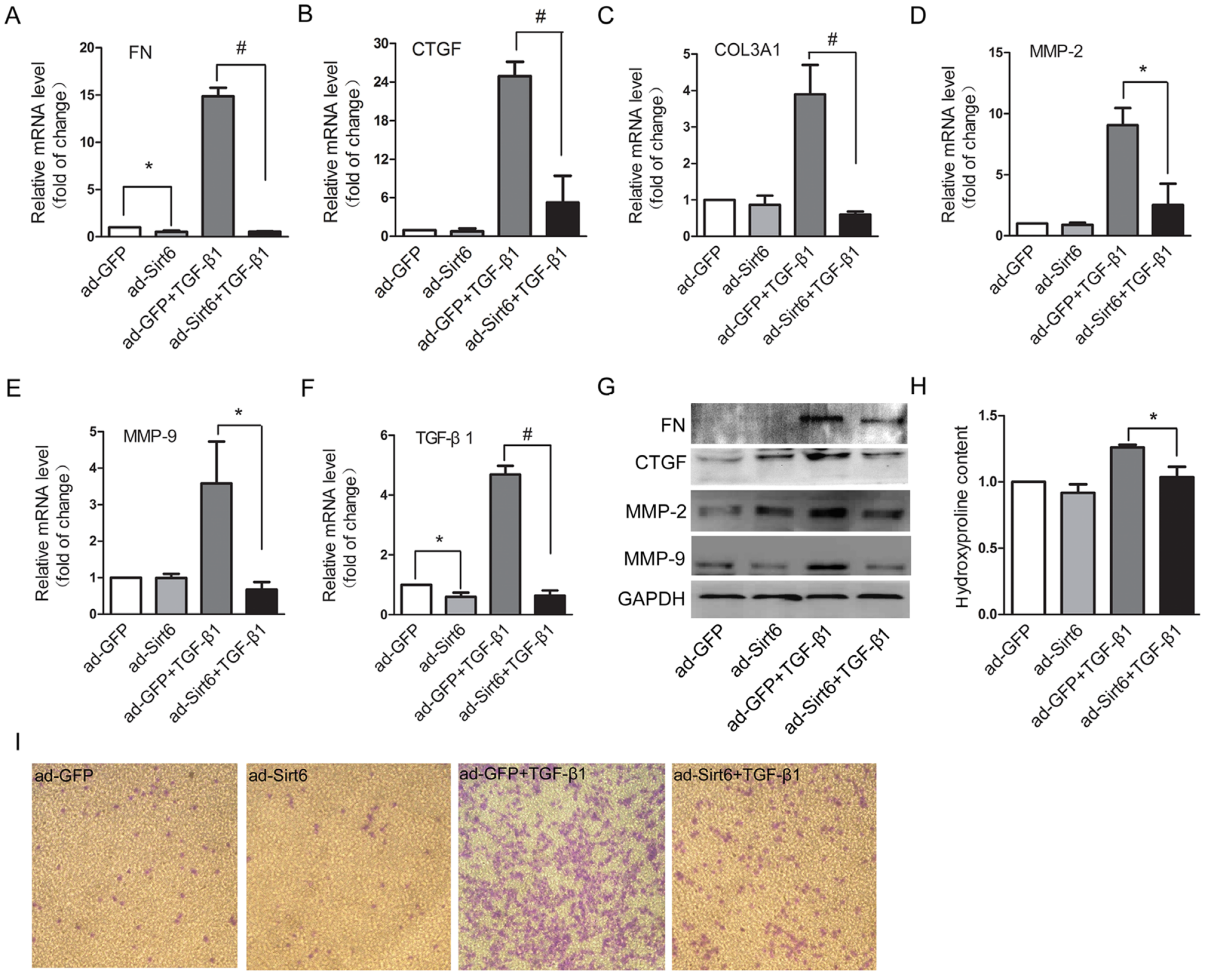
Sirt6 controls EMT-associated cell behaviors A549 cells were transfected with ad-GFP or ad-Sirt6 in the absence or presence of TGF-β1 (5 ng/ml) for 24 h. **(A-F)** Real-time PCR analysis of the mRNA levels of FN (A), CTGF (B), COL3A1 (C), MMP-2 (D), MMP-9 (E), and TGF-β1 (F). **(G)** Western blot analysis of the protein levels of FN, CTGF, MMP-2, and MMP-9. **(H)** The content of hydroxyproline in the supernatant was measured by hydroxyproline assay. **(I)** After the above treatment, A549 cells were seeded in the transwell chamber and cultured for 24 h. The transmigrating cells were stained and visualized by a microscope (magnification, 100 ×). All data represent the means ± SEM of three independent experiments. Compared with ad-GFP+TGF-β1 group, ^*^P < 0.05; ^#^*P* < 0.01.

**Figure 4:**
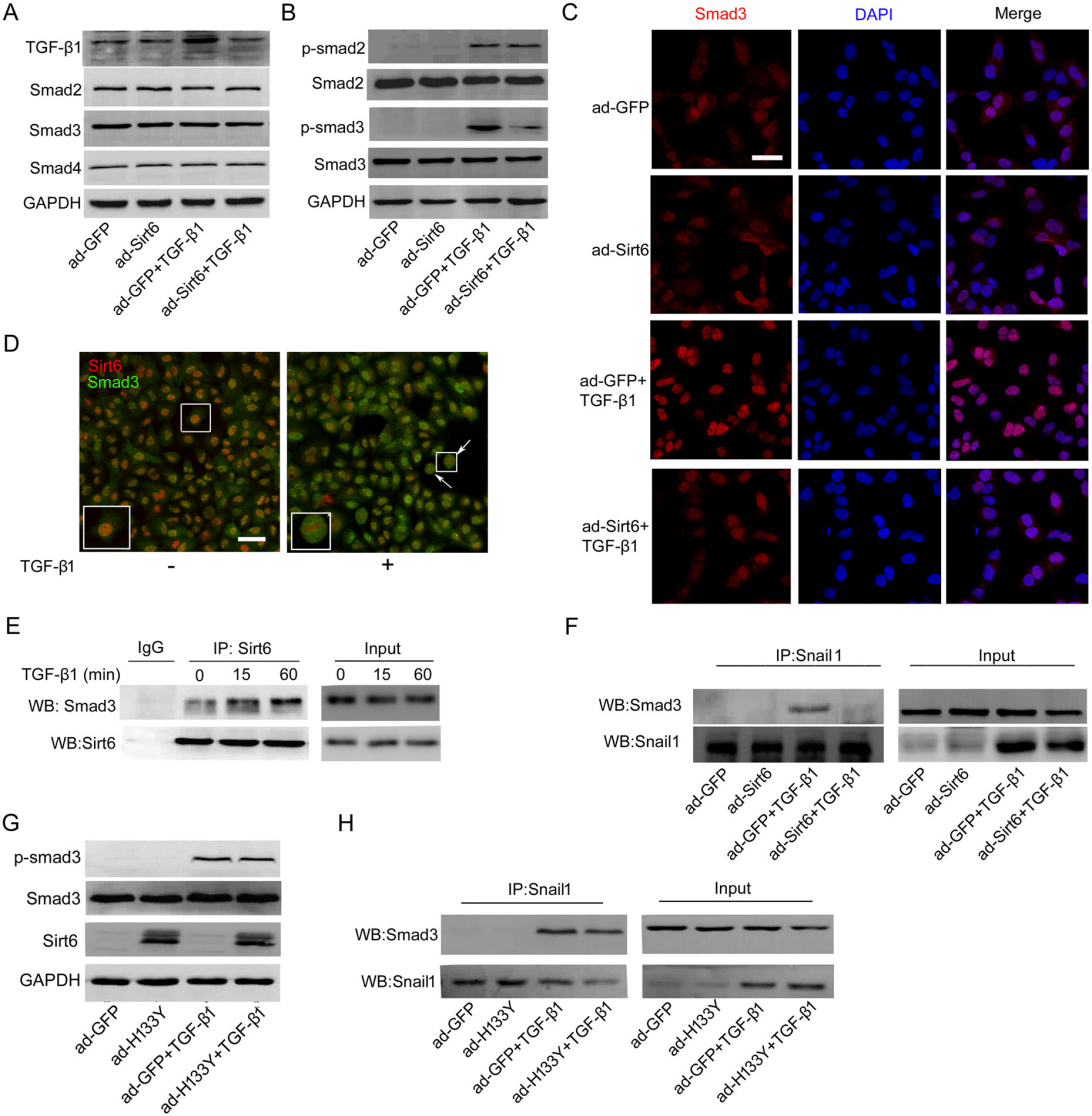
Sirt6 attenuates TGF-β1/Smad3 signaling **(A)** A549 cells were transfected with ad-GFP or ad-Sirt6 in the absence or presence of TGF-β1 (5 ng/ml) for 24 h, and the protein levels of TGF-β1, Smad2, Smad3, and Smad4 were measured by Western blot. **(B-C)** A549 cells were transfected with ad-GFP or ad-Sirt6 in the absence or presence of TGF-β1 (5 ng/ml) for 60 min. (B) The protein levels of p-Smad2 and p-Smad3 were measured by Western blot. (C) Representative images of the immunofluorescent staining of Smad3. Scale bar, 40 μm. **(D)** Representative images of double immunostaining of Sirt6 and Smad3. Scale bar, 50 μm. **(E)** A549 cells were treated with or without TGF-β1 (5 ng/ml) for 15 and 60 min. Total protein was subjected to co-IP with anti-Sirt6 antibody. **(F)** A549 cells were transfected with ad-GFP or ad-Sirt6 in the absence or presence of TGF-β1 (5 ng/ml) for 24 h, followed by co-IP with anti-Snail1 antibody. A549 cells were transfected with ad-GFP or ad-H133Y in the absence or presence of TGF-β1 (5 ng/ml) followed by **(G)** Western blot to measure the protein levels of p-Smad3 after 60 min treatment and **(H)** co-IP with anti-Snail1 antibody after 24 h treatment.

Lung epithelial cells appear to be the main target of lung injury in IPF. Following persistent lung injury, epithelial cells not only undergo phenotypic change but also undergo functional change that are characterized by synthesis and secretion of a series of profibrotic factors, which in turn contributes to EMT and IPF [20, 21]. Thus, we examined whether inhibition of EMT by Sirt6 contributed to abrogated fibrotic response. TGF-β1 dramatically upregulated the mRNA and protein levels of FN, CTGF, MMP-2, MMP-9, and TGF-β1, and the mRNA level of COL3A1, all of which were ameliorated by overexpression of Sirt6 (Figure 3A-3G, Figure 4A). Hydroxyproline is the most specific amino acid in collagen. The increased content of hydroxyproline elicited by TGF-β1 was also reversed by Sirt6 overexpression (Figure 3H). Epithelial cells undergoing EMT acquire migratory ability, a phenotype associated with mesenchymal cells [22]. We showed that forced expression of Sirt6 almost completely abolished TGF-β1-induced migratory behavior (Figure 3I). Therefore, Sirt6 controls TGF-β1-induced mesenchymal cell behaviors.

### TGF-β1/Smad3 signaling is involved in Sirt6-mediated EMT inhibition

Next, we determined the molecular mechanisms by which Sirt6 suppresses TGF-β1-induced EMT in A549 cells. TGF-β/Smads is the primary signaling pathway regulating EMT. Upon TGF-β1 stimulation, Smad2 and Smad3 are phosphorylated and form trimers with Smad4, and the complex translocate into the nucleus, where they associate and cooperate with other transcription factors to modulate target gene transcription [23]. We first assessed whether Sirt6 can ameliorate TGF-β/Smads pathway. As shown in Figure 4A, the marked increase of TGF-β1 protein level elicited by TGF-β1 treatment was blunted upon Sirt6 overexpression. Interestingly, there was no significant difference in the protein expression of Smad2, Smad3, and Smad4 between ad-GFP and ad-Sirt6 transfected cells in the presence or absence of TGF-β1 (Figure 4A). However, Sirt6 overexpression severely impaired phosphorylation of Smad3, but not Smad2, in response to TGF-β1 stimulation (Figure 4B). TGF-β1-induced nuclear translocation of Smad3 was also blocked by forced expression of Sirt6 (Figure 4C). These results suggested that Sirt6 may impair EMT through inhibiting TGF-β1/Smad3 signaling.

We observed that Smad3 translocated into the nucleus and co-located with Sirt6 in the nucleus upon TGF-β1 treatment by double immunostaining (Figure 4D), suggesting a potential interaction between Sirt6 and Smad3. To further gain insight into how Sirt6 suppresses Smad3, co-immunoprecipitation (co-IP) assay was performed and revealed that Sirt6 directly interacted with Smad3 (Figure 4E).

During TGF-β1-induced EMT process, Smads induce gene reprogramming via directly activating EMT-related transcription factors including Snail1 and ZEB family, and then cooperate with these factors to control transcription of EMT- and fibrosis-associated genes [11, 24, 25]. To systematically elucidate the underlying mechanisms by which Sirt6 impaired EMT process, we employed co-IP analysis and found that Sirt6 overexpression blocked TGF-β1 induced Snail1-Smad3 interaction (Figure 4F). However, mutant Sirt6 (H133Y) without histone deacetylase activity failed to repress Smad3 phosphorylation and Snail1-Smad3 binding (Figure 4G and 4H). Thus, the anti-EMT effects of Sirt6 are most likely caused by inactivating TGF-β1/Smad3 signaling, which is dependent on the deacetylase activity of Sirt6.

### Sirt6 attenuates EMT-related transcription factors

**Figure 5:**
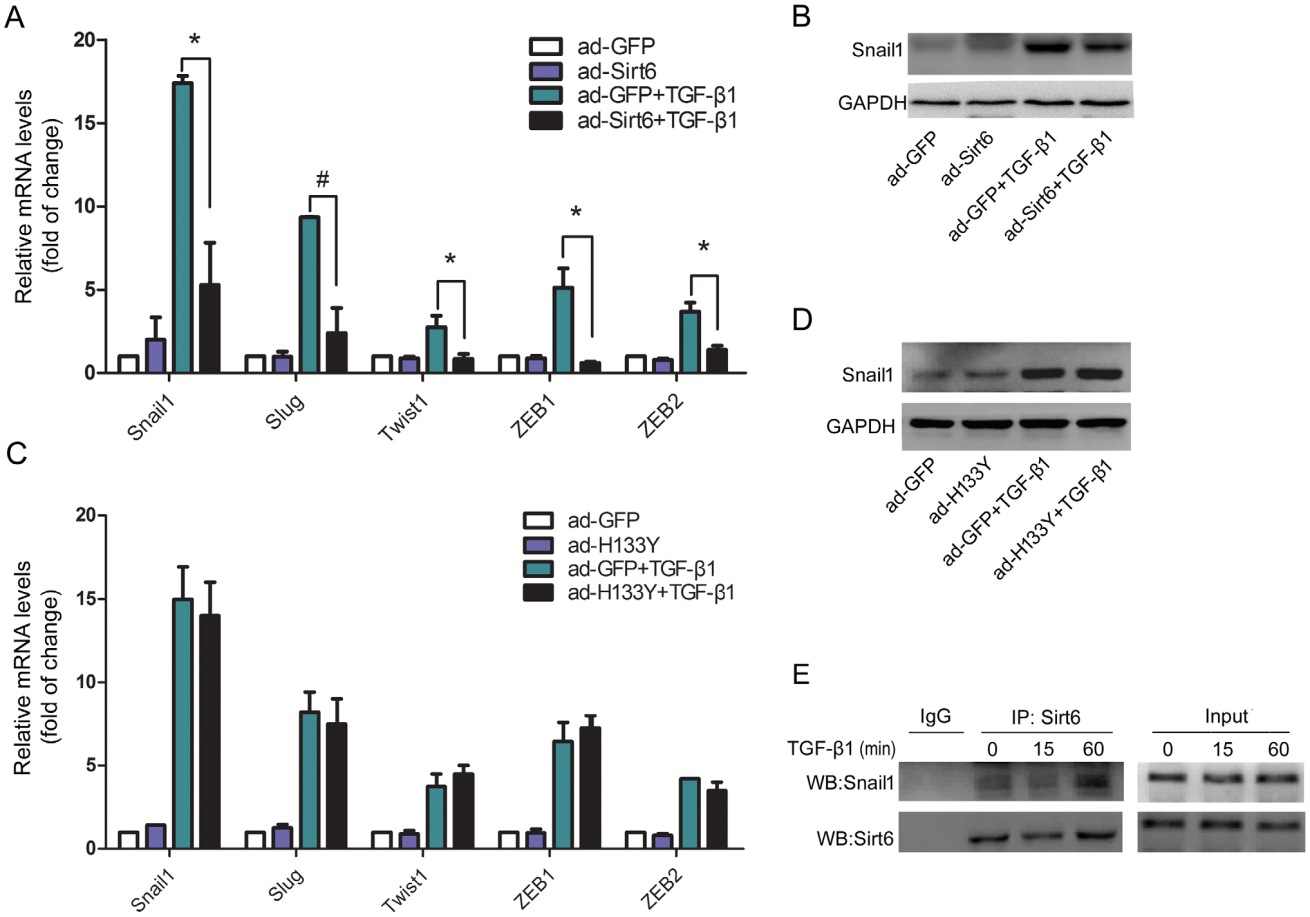
Sirt6 inhibits the expression of EMT-related transcription factors A549 cells were transfected with ad-GFP or ad-Sirt6 in the absence or presence of TGF-β1 (5 ng/ml) for 24 h. **(A)** Real-time PCR analysis of the mRNA levels of Snail1, Slug, Twist1, ZEB1, and ZEB2. **(B)** Western blot analysis of Snail1 protein. A549 cells were transfected with ad-GFP or ad-H133Y in the absence or presence of TGF-β1 (5 ng/ml) for 24 h. **(C)** Real-time PCR analysis of the mRNA levels of Snail1, Slug, Twist1, ZEB1, and ZEB2. **(D)** Western blot analysis of Snail1 protein. **(E)** A549 cells were treated with TGF-β1 (5 ng/ml) for 15 and 60 min, and total protein was co-immunoprecipitated with anti-Sirt6 antibody. All data represent the means ± SEM of three independent experiments. Compared with ad-GFP+TGF-β1 group, ^*^P < 0.05; ^#^*P* < 0.01.

We next investigated the effects of Sirt6 on the expression of EMT-related transcription factors. Sirt6 overexpression significantly reduced the transcription levels of Snail1, Slug, Twist1, ZEB1, and ZEB2 in TGF-β1-treated A549 cells (Figure 5A). Additionally, the protein level of Snail1 was also abrogated by gain of Sirt6 in TGF-β1-treated A549 cells (Figure 5B). However, mutant Sirt6 (H133Y) failed to abrogate the mRNA levels of these transcription factors (Figure 5C) and Snail1 protein expression (Figure 5D). Additionally, we performed co-IP assay and found that Sirt6 interacted with Snail1 (Figure 5E). In summary, these results suggest that Sirt6 inhibited EMT-related transcription factors in a catalytic activity-dependent manner.

### EMT is inhibited in Sirt6^AAV^ mice in response to bleomycin

**Figure 6:**
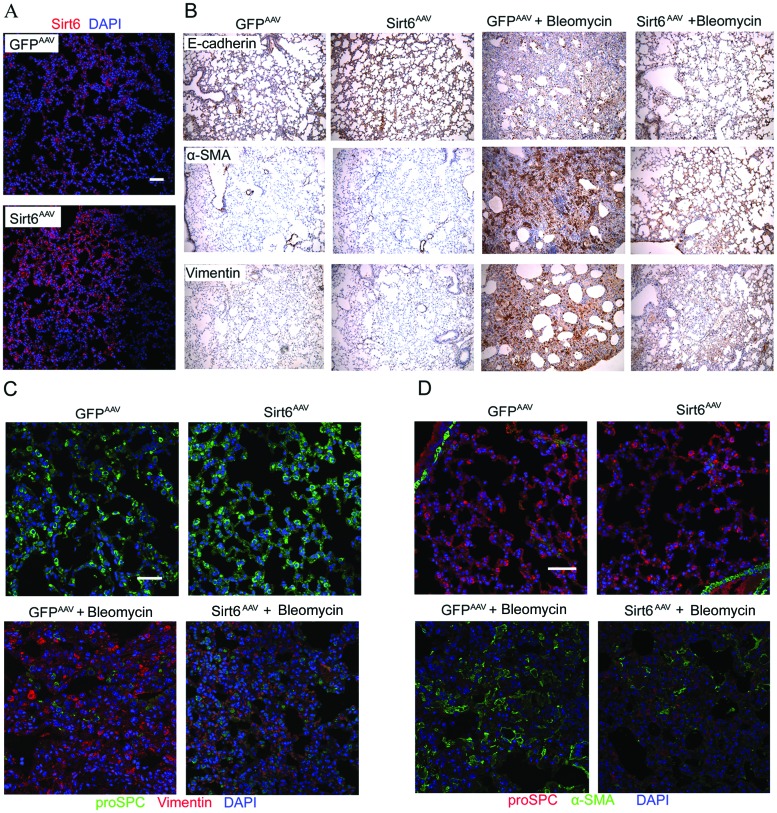
AAV-Sirt6 delivery ameliorates bleomycin-induced pulmonary EMT **(A)** Immunofluorescent analysis of lung sections to determine the efficiency of Sirt6 delivery after mouse intratracheal injection of AAV-Sirt6 (2×10^11^ vg) for 21 days. Scale bar, 50 μm; n=3 mice per group. **(B-D)** GFP^AAV^ and Sirt6^AAV^ mice were treated with 2 mg/kg bleomycin for 21 days. Lung sections were analyzed with IHC with antibodies against E-cadherin, α-SMA, and vimentin (magnification, 100×). n = 6 mice per group (B). Immunofluorescent staining was performed to measure proSPC and vimentin (C), and proSPC and α-SMA (D) expression in mouse lung. Scale bar, 50 μm; n = 3 mice per group.

To confirm the anti-EMT effect of Sirt6 *in vivo*, we constructed Sirt6^AAV^ mice by intratracheal injection of adeno-associated virus (AAV)-Sirt6. AAV vector, an appropriate vector for gene transfers, is small and non-pathogenic compared with adenovirus vector. Importantly, they are capable of promoting persistent gene expression in multiple somatic tissues of animals compared with adenovirus and currently being tested in several clinical trials [26]. Initially, we performed immunofluorescent staining to confirm AAV-mediated delivery of Sirt6 in mouse lung. As shown in Figure 6A, Sirt6 successfully reached alveolar walls after intratracheal injection of AAV-Sirt6. Bleomycin markedly promoted EMT phenotype in GFP^AAV^ mice, as shown by enhanced vimentin and α-SMA staining but diminished E-cadherin in AECs detected by IHC staining. In contrast, this effect was significantly blunted in Sirt6^AAV^ mice (Figure 6B). In addition, we performed double immunostaining to further investigate whether Sirt6 was capable of preventing AECs undergoing EMT *in vivo*. As shown in Figure 6C and 6D, there were an increase in proSPC, a type II AECs marker, and a decrease in vimentin and α-SMA staining in Sirt6^AAV^ mice compared with GFP^AAV^ littermate mice in response to bleomycin treatment. Taken together, delivery of Sirt6 to lung abrogated bleomycin-induced EMT-like phenotype.

### Lung fibrosis is reduced in Sirt6^AAV^ mice in response to bleomycin

**Figure 7:**
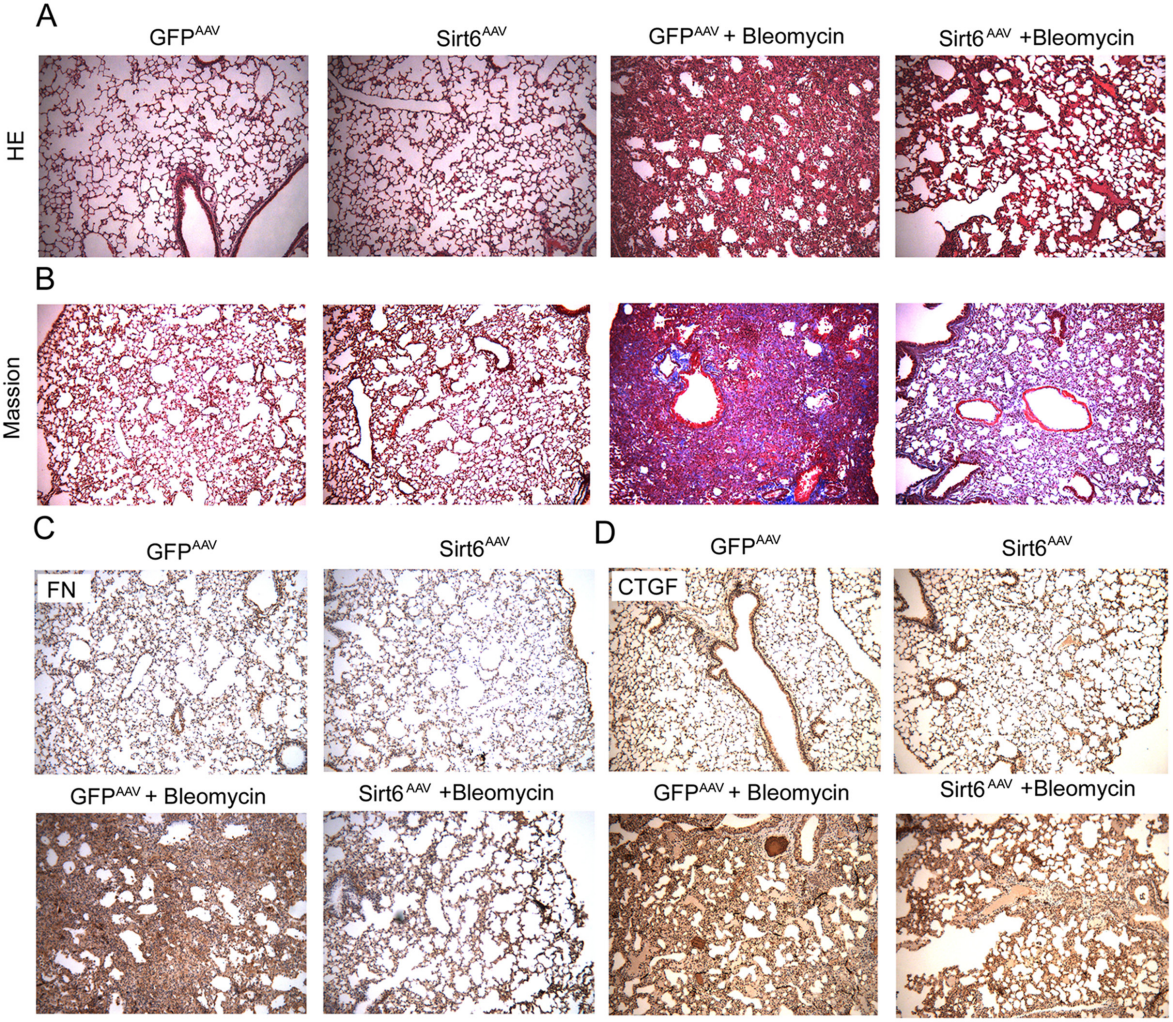
AAV-Sirt6 delivery protects bleomycin-induced pulmonary fibrosis GFP^AAV^ and Sirt6^AAV^ mice were treated with 2 mg/kg bleomycin for 21 days. Sections of pulmonary tissues were harvested at day 21 and subjected to HE **(A)**, Masson’s trichrome staining **(B)**, and IHC staining for FN **(C)** and CTGF **(D)** (magnification, 100 ×). n = 6 mice per group.

Several lines of evidence indicated that EMT is involved in the pathogenesis of lung fibrosis. Therefore, we speculated that inhibition of EMT by Sirt6 may contribute to eased lung fibrosis. After bleomycin instillation, mice displayed pulmonary parenchymal damage, collapsed alveoli, and thicker alveolar membrane that were determined by HE staining (Figure 7A); Masson's trichrome staining of collagen showed severe collagen deposition in lung mesenchyme (Figure 7B). These bleomycin-induced fibrotic phenomenon were abolished in Sirt6^AAV^ mice. Moreover, IHC analysis revealed that bleomycin-increased FN (Figure 7C) and CTGF (Figure 7D) were also ameliorated in Sirt6^AAV^ mice. Overall, these findings indicated that AAV-mediated lung-targeted delivery of Sirt6 alleviated pulmonary fibrosis.

## DISCUSSION

In the present study, we demonstrated that gain of Sirt6 is sufficient to protect A549 cells from mesenchymal phenotype and EMT-like cell behaviors induced by TGF-β1. Lung targeted delivery of Sirt6 by AAV rescued lung epithelial cell injury and fibrosis induced by bleomycin. Mechanistically, we found that Sirt6 inhibited EMT through repressing TGF-β1/Smad3 signaling pathway, Smad3-Snail1 interaction, and EMT-associated transcription factors, all of which required Sirt6 deacetylase activity.

In the first set of experiments, we found that Sirt6 was upregulated by both TGF-β1 *in vitro* and bleomycin *in vivo*. Sirt6 has been found to be induced in response to various stimuli, such as H_2_O_2_ [27], paraquat [27], and TGF-β1 [28], and it protects against DNA damage and cell senescence. Therefore, upregulation of Sirt6 under stress may be a compensatory mechanism to maintain the functions and homeostasis of the cells in early stage of diseases. As the dose and treatment time increased, the cells entered a decompensatory stage with no change of Sirt6.

There are increasing evidences suggesting that EMT functions in the pathological process of IPF. Better understanding of the molecular mechanism of EMT is crucial for the development of novel therapeutic approach for patients with IPF. Albeit Sirt6 protects bronchial epithelial cells from TGF-β1-and cigarette smoke extract-induced senescence [28, 29], the influence of Sirt6 on EMT process has not been described previously. A major finding of this study is that Sirt6 negatively regulated EMT phenotype both *in vitro* and *in vivo*. Interestingly, EMT process is a reversible biological process which is a form of cellular plasticity that refers to redifferentiation of mesenchymal cells to epithelial cells [25]. Notably, Sirt6 also participates in differentiation of various of cell types, such as squamous cells [30] and embryonic stem cells [31]. Unexpectedly, loss of Sirt6 had no effect on EMT process. One plausible explanation is that there are seven members in sirtuin family. Once Sirt6 is knocked down, other members may compensate for the loss of Sirt6 function [32]. To date, the strategies targeting EMT-related fibrosis are mainly focused on preventing the production of EMT-originated myofibroblats, removing active myofibroblats, and de-differentiating myofibroblats to epithelial cells. Therefore, the present study has a significant implication that proper regulation of the cellular plasticity by Sirt6 may be an effective therapeutic method for IPF.

Previous studies indicated that both liver and heart exhibited increased fibrosis in Sirt6 mutant mice via activating IGF/Akt [17] and c-Jun signaling [19], respectively. Consistent with the anti-fibrotic notion, we also found that Sirt6 overexpression inhibited lung fibrosis, as evidenced by diminished synthesis and secretion of fibrotic factors and ECM, and EMT-like cell behaviors. We speculated that impaired EMT process *in vivo* and *in vitro* by Sirt6 may contribute to reduced accumulation of myofibroblasts and eventually abrogated fibrotic response.

**Figure 8:**
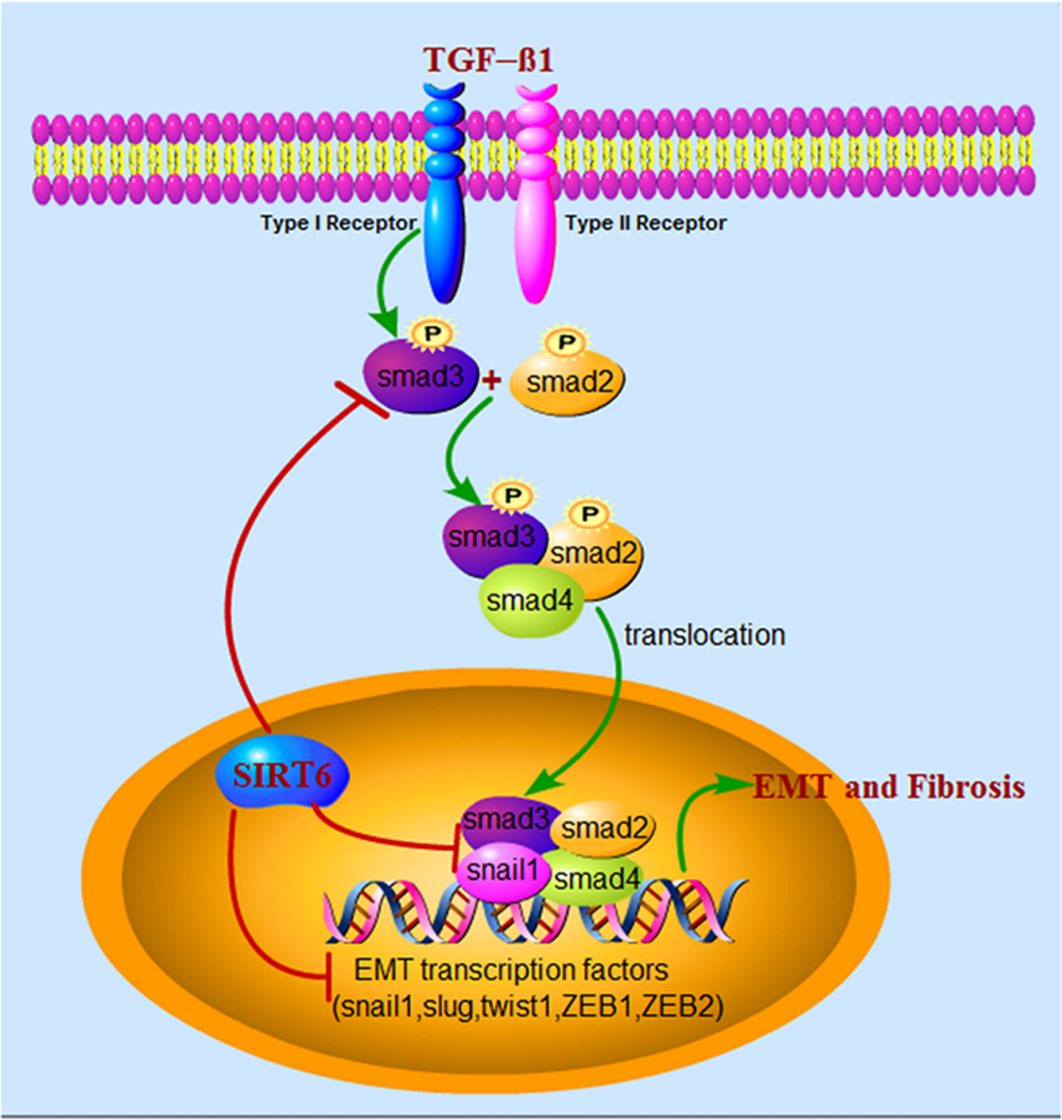
Schematic illustration of the molecular mechanisms of Sirt6 in regulating EMT of lung epithelial cells during IPF Upon TGF-β1 stimulation, Smad2 and Smad3 are phosphorylated by type I receptor, then form complex with Smad4 and eventually translocate to the nucleus, where they cooperate with EMT-related transcription factors, for example Snail1, to regulate the transcription of TGF-β1-responsive genes. Sirt6, a histone deacetylase located in the nucleus, inhibits EMT mainly through three different means. (1) Decreases phosphorylation of Smad3, and thereby inhibit nuclear translocation of Smad3 and binding of Smad3 and Snail1 or other EMT transcription factors; (2) Directly binds to Smad3, and may deacetylate H3K9 at the promoter of Smad3 target genes to suppress their expression; (3) Directly diminishes the expression of EMT-related transcription factors.

We further elaborated the mechanisms involved in EMT regulation by Sirt6 (Figure 8). Sirt6 inhibits EMT mainly through three different means. (1) Decreases phosphorylation of Smad3, and thereby inhibit nuclear translocation of Smad3 and binding of Smad3 and Snail1 or other EMT transcription factors; (2) Directly binds to Smad3, and probably deacetylate H3K9 at the promoter of Smad3 target genes to suppress their expression; (3) Directly diminishes the expression of EMT-related transcription factors.

It is known that Sirt6 inactivates NF-κB [33], IGF-Akt [17], and Wnt signaling pathways [34]. To the best of our knowledge, the effect of Sirt6 on TGF-β1/Smad3 signaling has not been previously described. Our result indicates that Sirt6 directly binds to Smad3. However, how sirt6, a histone deacetylase, modulates phosphorylation of Smad3 is still unknown. We hypothesize that Sirt6 mediated Smad3 phosphorylation through an indirect manner. This may occur through inhibition of endogenous TGF-β1 synthesis by Sirt6 overexpression and subsequently decreasing TGF-β1-induced phosphorylation of Smad3. Therefore, Sirt6 inhibits Smad3 phosphorylation possibly via decreasing the production of TGF-β1.

Smad3 promotes and cooperates with EMT-associated transcription factors which facilitates development of EMT [11, 24, 35]. It has been demonstrated that Sirt6 interacted with NF-κB [33] or Hif-1α [36], and deacetylated H3K9 at the promoter of their target genes to suppress transcription of these genes. Thus, a possible explanation is that Sirt6 is recruited to these genomic loci via a physical interaction with Smad3, deacetylates histone H3K9 and thereby diminished expression of EMT-related transcription factors.

In summary, we identified that Sirt6 suppressed EMT phenotype *in vivo* and *in vitro*. The anti-EMT effect of Sirt6 may partially through inhibiting TGF-β1/Smad3 signaling pathway. Therefore, optimal level of TGF-β1/Smad3 signaling or EMT suppression achieved via Sirt6 activation is a potential effective medical intervention under IPF context. Future study aims to develop effective and safe agonist or target gene delivery method for Sirt6 to treat IPF. Furthermore, in the following study, we will seek to address the possible epigenetic regulation of Smad3 target genes by Sirt6 in A549 cells.

## MATERIALS AND METHODS

### Ethics statement, experimental lung fibrosis model and AAV delivery

Investigation has been conducted in accordance with the ethical standards and according to the Declaration of Helsinki as well as national and international guidelines. The study has also been approved by the Institutional Animal Care and Use Committee of Tongji Medical College (Wuhan, China). Male C57BL/6 mice (8-week-old; 20∼30 g) were obtained from Hubei Research Center of Laboratory Animals. AAV expressing GFP or Sirt6 (Vigene Bioscience, Jinan, China) was delivered to mouse lung by intratracheal injection with 50 μl PBS containing 2×10^11^ vg per mouse, and termed as GFP^AAV^ and Sirt6^AAV^ mice, respectively. 2 mg/kg bleomycin (Nippon Kayaku, Tokoyo, Japan) was intratracheally injected one week later. Mice were sacrificed 3 weeks after bleomycin administration.

### Cell culture and transfection

A549 cells have been widely used to study pulmonary EMT during the pathogenesis of IPF [15, 37]. The cells were purchased from the Cell Bank of Chinese Academy of Sciences (Shanghai, China) and cultured in DMEM containing 10% fetal bovine serum (Gibco, Grand Island, USA) at 37°C in a humidified chamber with 5% CO_2_. The cells were transfected with adenovirus vector encoding Sirt6 (ad-Sirt6) obtained from Vigene Bioscience. Negative control (NC) siRNA and Sirt6 siRNA were purchased from GenePharma (Shanghai, China). The sequences of siRNAs are shown in Supplementary Materials (Supplementary Table 1). Catalytic inactive Sirt6 (ad-H133Y) is a kind gift of prof. Depei Liu (Peking Union Medical College).

### Real-time RT-PCR analysis

Total RNA was extracted from A549 cells and converted into cDNA using Reverse Transcription kit (TaKaRa, Kyoto, Japan). The mRNA levels were detected with SYBR using real-time PCR system (Applied Biosystems, Foster City, USA). Primers for real-time RT-PCR are listed in Supplementary Table 2.

### Western blot

Treated A549 cells were lysed by RIPA lysis buffer and total protein was extracted. Primary antibodies for Sirt6, Smad2, Smad3, Smad4, p-Smad2, and p-Smad3 were purchased from Cell Signaling Technology (Danvers, USA). CTGF, TGF-β1, vimentin, α-SMA, and Smad3 were obtained from Abcam (Cambridge, UK). E-cadherin and FN were purchased from Santa Cruz Biotechnology (Santa Cruz, USA).

### Hydroxyproline measurement

The content of hydroxyproline in A549 cell culture supernatant was measured by a commercial kit (Nanjing Jiancheng Bioengineering Institute, Nanjing, China) according to the manufacturer’s instructions.

### Immnofluorescent staining

A549 cells cultured on 6-well chamber slides or paraffin section of lung tissues were fixed in 4% paraformaldehyde, and permeabilized with 0.1% Triton X-100. The slides or sections were then blocked with 10% goat serum at room temperature for 30 minutes. Subsequently, the slides or sections were incubated with primary antibodies at 4°C overnight, followed by incubation with Alexa Fluor 594 or Alexa Fluor 488 secondary antibodies (Invitrogen, Carlsbad, USA) for 1 hour at room temperature. The slides and sections were counterstained with DAPI and then examined by confocal microscopy (Olympus, Tokoyo, Japan). Primary antibodies include rabbit anti-E-cadherin, rabbit anti-vimentin, rabbit anti-α-SMA, and rabbit anti-Smad3 from CST, and rabbit anti-proSPC, mouse anti-Smad3, mouse anti-vimentin, and mouse anti-α-SMA from abcam.

### Co-immunoprecipitation

50 μl protein G magnetic beads (Invitrogen) were washed with 200 μl antibody binding&washing buffer. After removal of the supernatant, the beads were collected and incubated with rabbit anti-Sirt6 or rabbit anti-Snail1 antibodies (abcam) by gentle rotation for 10 min. Subsequently, the antibody-beads complex was incubated with 400 μg total protein that was extracted with NP-40 lysis buffer via vortexing for 15 min. The supernatant was removed and the antibody-protein-beads complex was washed 3 times using 200 μl washing buffer. The supernatant was removed again and the antibody-protein-beads complex was gently resuspended with 20 μl elution buffer for 2 min. The sample was separated and subjected to Western blot analysis.

### Transwell assay

Following the indicated treatment, the cells were trypsinized and seeded in the upper chamber (Corning, Corning, USA) with serum free medium at a density of 1×10^5^ cells/well. DMEM containing 10% FBS was added to the lower chamber. After 24 h, non-migrating cells were removed with a swab, and migrating cells were fixed with 4% paraformaldehyde and stained with 0.1% crystal violet.

### Histological analysis

Lung tissues were fixed in 4% paraformaldehyde and then embedded in paraffin before being cut into 4 μm thick sections. Paraffin-embedded sections of mouse tissues were stained with Masson’s trichrome and HE, and IHC analysis was performed as previously described [38]. Paraffin-embedded sections were stained with antibodies specific for E-cadherin, vimentin, α-SMA, FN, and CTGF (abcam). Representative images were captured using a microscope (Olympus).

### Statistical analysis

Data are presented as the means ± SEM. Statistical analysis was performed with unpaired Student’s *t*-test between two groups and differences among groups were tested by one-way ANOVA with Tukey’s *post hoc* test. Analysis was performed using SPSS 17.0 software (IBM, USA). In all cases, differences were considered statistically significant with *P* < 0.05 and statistically highly significant with P < 0.01.

## SUPPLEMENTARY MATERIALS FIGURES AND TABLES



## Abbreviations

IPF: idiopathic pulmonary fibrosis
AECs: alveolar epithelial cells
TGF-β1: transforming growth factor 1
ECM: extracellular matrix
EMT: epithelial to mesenchymal transition
FN: fibronectin
α-SMA: smooth muscle actin
AAV: adeno-associated virus
