# Supplementary material for: Sirtuin 6 inhibits epithelial to mesenchymal transition during idiopathic pulmonary fibrosis via inactivating TGF-β1/Smad3 signaling
Source: Oncotarget. 2017 May 9;8(37):61011–24. doi: 10.18632/oncotarget.17723 (PMC5617402; doi:10.18632/oncotarget.17723)
Supplement: Supplementary file 1 [file oncotarget-08-61011-s001.pdf]

Sirtuin 6 inhibits epithelial to mesenchymal transition during idiopathic pulmonary fibrosis via inactivating TGF-β1/Smad3 signaling

SUPPLEMENTARY MATERIALS

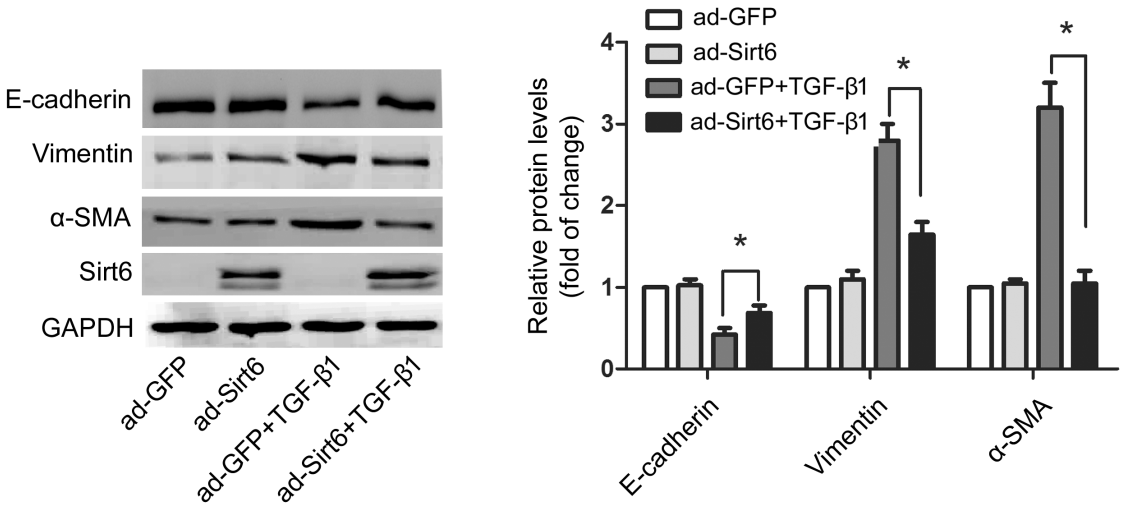

Supplementary Figure 1: Overexpression of Sirt6 inhibits EMT phenotype induced by TGF-β1 in primary mouse alveolar epithelial cells. Primary mouse alveolar epithelial cells were transfected with ad-GFP or ad-Sirt6 in the absence or presence of TGF-β1 (5 ng/ml) for 24 h. Western blot analysis of the protein levels of E-cadherin, vimentin, and α-SMA was performed. Compared with ad-GFP+TGF-β1 group, $^{*}P < 0.05$.

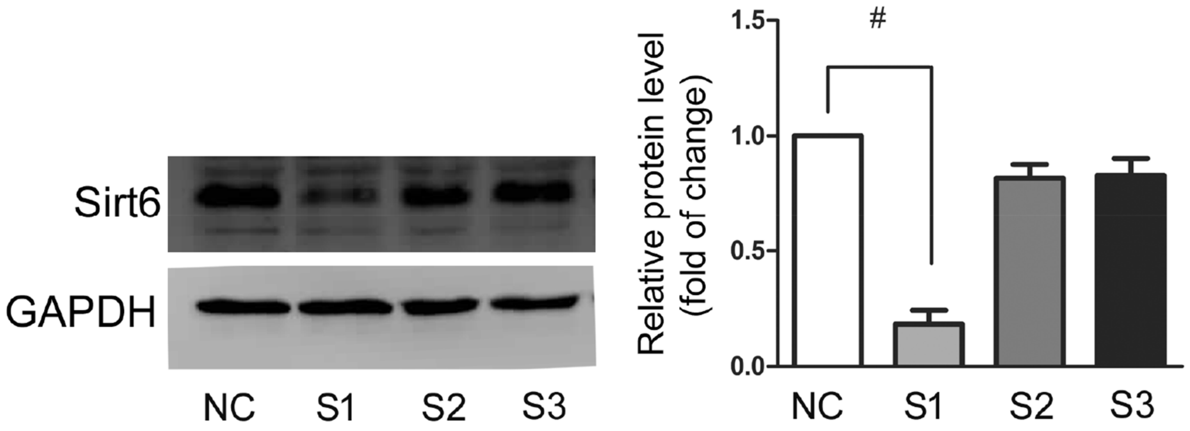

Supplementary Figure 2: Sirt6 was efficiently knocked down using siRNA for Sirt6. Three independent siRNAs targeted Sirt6 (marked as S1, S2, and S3), as well as negative control (NC), were transfected into A549 cells. The protein expression of Sirt6 was measured by Western blotting. [#]*P*<0.05 as compared with NC siRNA.

Supplementary Table 1: Sequences of siRNA oligos for Sirt6

siRNA	Sequence
S1	Sense:5' -AGCGGAAGGUGUGGGAACUTT-3'<br>anti-sense: 5' -AGUUCCCACACCUUCCGCUTT-3'
S2	Sense:5'-UCCAUCACGCUGGGUACAUTT-3'<br>anti-sense :5' -AUGUACCCAGCGUGAUGGATT-3
S3	Sense:5' -UCAUGACCCGGCUCAUGAATT-3'<br>anti-sense: 5' -UUCAUGAGCCGGGUCAUGATT-3'

Supplementary Table 2: Primer sets used for real-time PCR

Target gene	Sequence
E-cadherin	F: 5′- GCCGAGAGCTACACGTTCAC-3′
	R:5′- CACACCATCTGTGCCCACTT-3′
Vimentin	F: 5′- GGACCAGCTAACCAACGACA-3′
	R: 5′- AAGGTCAAGACGTGCCAGAG-3′
α-SMA	F: 5′- ACTGCCTTGGTGTGTGACAA-3′
	R: 5′- CACCATCACCCCCTGATGTC-3′
ZEB1	F: 5′-GAAAATGAGCAAAACCATGATCCTA-3′;
	R: 5′-CAGGTGCCTCAGGAAAAATGA-3′
ZEB2	F: 5′- AAGGAGCAGGTAATCGCAAG-3′.
	R: 5′-TTTGGGCACTCGTAAGGTTT-3′.
Snail1	F: 5′-TTTACCTTCCAGCAGCCCTA-3′
	R: 5′-GACAGAGTCCCAGATGAGCA-3′
Slug	F:5′-GCTACCCAATGGCCTCTCTC-3′
	R: 5′-CTTCAATGGCATGGGGGTCT-3′
Twist1	F:5′- TCGGACAAGCTGAGCAAGAT-3′
	R:5′-CCATCCTCCAGACCGAGAAG-3′
FN	F:5′-CCAGTCCCGAAGGCACTAC-3′
	R:5′-CTCTCGGGAATCTTCTCTGTCA-3′
CTGF	F:5′- GCCTCTTCTGTGACTTCGGC-3′
	R:5′-CTCTGGAAGGACTCTCCGCT-3′
COL3A1	F:5′-ACGGAAACACTGGTGGACAG-3′
	R:5′-GAAGCTCGGCTGGAGAGAAG-3′
MMP-2	F:5′- TATGGCTTCTGCCCTGAGAC-3′
	R:5′- CACACCACATCTTTCCGTCA-3′
MMP-9	F:5′-GAAGCTCGGCTGGAGAGAAG-3′
	R:5′-TTTGAGTCCGGTGGACGATG-3′
Sirt6	F:5′-GGAAAAGGGTGTGAAGAGGCAGGCT-3′
	R:5′-GGAAAAGGGTGTGAAGAGGCAGGCT-3′
GAPDH	F:5'-AGGAGTAAGAAACCCTGGAC-3'
	R:5'-CTGGGATGGAATTGTGAG-3'
